# Asthenopia

**Published:** 1877-04

**Authors:** 


### ASTHENOPIA.

This is a difficulty of vision in which the letters, in reading, become blurred and seem to run one into the other, with a bright halo about them, sometimes one line overlapping the other, giving rise to double vision. In looking at objects near by, for a little while, they also appear double, and reading for any considerable time, produces pain in and about the eyes, over the orbit and in the temples. If the eyes are rested a while, the reading may be resumed, but in a few minutes the blur and indistinctness returns, while the eyes become red, watery and painful.

These symptoms are produced by a weakness of the internal straight muscles, that turn the eye inward—a sort of paralysis of the muscles. It will be observed, while engaged in the act of reading, with the book held from ten to fifteen inches from the face, that the eyes are turned slightly inward toward the nose. The closer the book is held to the face, the more apparent becomes this convergence of the eyes. Now, if from any cause, the eyes do not incline precisely alike, the image made in each eye will not fall upon corresponding points on each retina, the result being that, both images are not blended into one, and a confused impression of the object is the result, giving rise to the symptoms first mentioned. To produce this result, in a familiar manner, you have only to fix your attention upon an object in the room, and then, with your finger, gently press your eye down or up in its socket, when instantly the object appears double and confused. That which happens here designedly, occurs when any of the four straight muscles that are located on each side and at the top and bottom of the eye-ball lose any of their full power to move the eye in the direction of their respective locations.

The *internal rectus*—as the muscle is named that turns the eye toward the nose, is the one most frequently found weakened, for the reason that it is the one most used in bringing the eye to the necessary position for scrutinizing an object near the face, as in reading, sewing and the like. When this muscle loses its power, through over-work or from sickness or other natural cause, the eyes deviate—a blurred and double image occurs, and the patient is said to have *muscular asthenopia.*

If a patient so afflicted is directed to look at your finger, while you hold it at twelve inches from his face; you will find that as you bring the finger closer to his eye—he looking at it steadily the while, there will be a point at about five or six inches distant, when the affected eye will begin to grow unsteady, wavering, and will gradually or perhaps suddenly turn outward. This test will at once reveal the eye affected, and determine which muscle is the weak one, for, if the eye turns outward, the internal rectus is the faulty one, if inward, the external. If the faulty eye be now covered so as to exclude it from participation in reading, it will be found to follow the direction of the strongest muscle and will squint outward or inward depending upon which side the weak muscle is. In order to avoid diplopia or double vision, a great effort is made by the patient to overcome the trouble by frowning and other muscular exertion, and in this manner brings on congestion and pain.

After a while, all control over the eye is lost, when it turns gradually outward and remains so, producing an outward squint of more or less degree, while the image is suppressed and the sound eye used exclusively. From want of exercise, the faulty eye soon becomes almost wholly blind, and the patient depends entirely upon the opposite one for all his work.

Asthenopia is easily remedied in the beginning, by use of spectacles. But the glasses used are unlike anything found where spectacles are usually sold. No one but an ophthalmic surgeon can apply them. They are prisms, of the proper power to compensate for the divergence of the weak eye. A prisim has the power of refracting a ray of light towards its base—toward the thick portion of the glass, consequently, when applied to an out-turning eye, with its base next the nose, the light is deflected toward the base of the prism, so entering the faulty eye in the same relative direction as it does in the sound one, at once restoring the equilibrium between the two optics and producing normal vision.

By applying prisms of various strengths to the eye, the ophthalmic surgeon can measure accurately, the loss of power in the muscle, and where it is very great, cuts the external muscle which is weakened to a corresponding degree to its fellow —so remedying the trouble. Often, it is preferable to resort to this latter mode of cure, but this, as all matters involving the care of our eyes, should be entrusted to the judgment and skill of the ophthalmic surgeon.

